# Stochastic Variation in Expression of the Tricarboxylic Acid Cycle Produces Persister Cells

**DOI:** 10.1128/mBio.01930-19

**Published:** 2019-09-17

**Authors:** Eliza A. Zalis, Austin S. Nuxoll, Sylvie Manuse, Geremy Clair, Lauren C. Radlinski, Brian P. Conlon, Joshua Adkins, Kim Lewis

**Affiliations:** a Department of Biology, Antimicrobial Discovery Center, Northeastern University, Boston, Massachusetts, USA; b Biological Sciences, Pacific Northwest National Laboratory, Richland, Washington, USA; c Department of Microbiology and Immunology, University of North Carolina School of Medicine, Chapel Hill, North Carolina, USA; Louis Stokes Veterans Affairs Medical Center; KU Leuven; Brown University

**Keywords:** *Staphylococcus aureus*, bioenergetics, heterologous gene expression, metabolism, persistence, tolerance

## Abstract

Chronic bacterial infections are difficult to eradicate, though they are caused primarily by drug-susceptible pathogens. Antibiotic-tolerant persisters largely account for this paradox. In spite of their significance in the recalcitrance of chronic infections, the mechanism of persister formation is poorly understood. We previously reported that a decrease in ATP levels leads to drug tolerance in Escherichia coli, Pseudomonas aeruginosa, and Staphylococcus aureus. We reasoned that stochastic fluctuation in the expression of tricarboxylic acid (TCA) cycle enzymes can produce cells with low energy levels. S. aureus knockouts in glutamate dehydrogenase, 2-oxoketoglutarate dehydrogenase, succinyl coenzyme A (CoA) synthetase, and fumarase have low ATP levels and exhibit increased tolerance of fluoroquinolone, aminoglycoside, and β-lactam antibiotics. Fluorescence-activated cell sorter (FACS) analysis of TCA genes shows a broad Gaussian distribution in a population, with differences of over 3 orders of magnitude in the levels of expression between individual cells. Sorted cells with low levels of TCA enzyme expression have an increased tolerance of antibiotic treatment. These findings suggest that fluctuations in the levels of expression of energy-generating components serve as a mechanism of persister formation.

## Abstract (precis)

Persister cells are rare phenotypic variants that are able to survive antibiotic treatment. Unlike resistant bacteria, which have specific mechanisms to prevent antibiotics from binding to their targets, persisters evade antibiotic killing by entering a tolerant nongrowing state. Persisters have been implicated in chronic infections in multiple species, and growing evidence suggests that persister cells are responsible for many cases of antibiotic treatment failure. New antibiotic treatment strategies aim to kill tolerant persister cells more effectively, but the mechanism of tolerance has remained unclear until now.

## INTRODUCTION

Toxin-antitoxin (TA) modules and the stringent response have been proposed as mechanisms of antibiotic tolerance, based primarily on studies of Escherichia coli. However, these findings have recently been challenged (1–4). Similarly, we reported previously that knocking out all TA modules and (p)ppGpp synthases in Staphylococcus aureus had no effect on persister formation (5). In search of an alternative mechanism, we found that persister cells in a growing population express stationary cell marker genes, *cap5A* and *arcA*, coding for capsular polysaccharide synthesis and arginine deiminase, respectively. Importantly, expression of *cap5A* and *arcA* was induced by treatment with arsenate, which depletes ATP through a futile cycle: ADP-As to ADP plus As (spontaneous hydrolysis). This suggests that these markers actually respond to ATP decreases and that the rare stationary-phase-like cells in a growing population have low energy levels. These cells showed considerably higher antibiotic tolerance after being sorted out from a growing population, showing that they are persisters. Importantly, dropping ATP to stationary-phase levels with arsenate treatment in a growing culture recapitulates the persister level of a stationary-phase population, showing that low energy is sufficient for tolerance. If low ATP levels result in tolerance, then high ATP levels should have the opposite effect. Indeed, supplementation of tryptic soy broth (TSB) medium with glucose increased ATP levels significantly and resulted in a 100-fold reduction in persisters. We observed similar results linking ATP and persisters in a study of E. coli (2). On the basis of these findings, we proposed a “low-energy” mechanism of persister formation. This hypothesis provides a satisfactory explanation for the mechanism of drug tolerance. Bactericidal compounds kill by corrupting active targets, and when ATP is low, cells become tolerant of antibiotics. In a stationary-phase population of S. aureus, ATP levels are indeed low. The entire population is highly tolerant of antibiotics and is equivalent to persisters. However, how ATP levels might decrease in rare cells of a growing population remained unknown. We reasoned that random fluctuations in the levels of energy-generating components could lead to the presence of low-energy cells. Here we show that cells in a growing population that have low levels of expression of tricarboxylic acid (TCA) cycle enzymes are tolerant of killing by antibiotics.

## RESULTS

**FIG 1:**
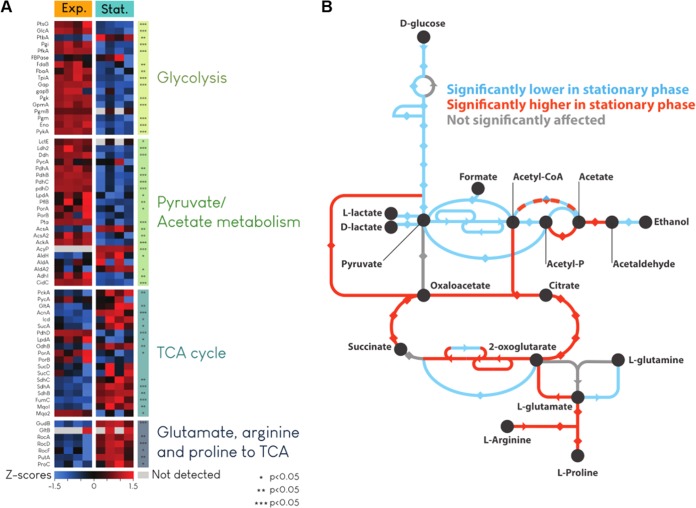
TCA enzyme abundance increases in the late growth phase. (A) Heat map showing enrichment analysis of proteins during exponential-phase (Exp.) and stationary-phase (Stat.) growth. (B) Map showing major steps in central metabolism for which significant changes in enzyme abundance were detected between exponential-phase growth and stationary-phase growth. Blue indicates decreased abundance in stationary phase; red indicates increased abundance in stationary phase. Glutamate-catabolizing and TCA cycle enzymes were detected in higher abundance in stationary phase, when glucose levels are known to be low. Four biological replicates were analyzed to determine relative levels of abundance for all conditions. Gene Ontology and KEGG identifiers were extracted from UniprotKB. Protein abundance significance was determined using Student’s *t* test. Fisher’s exact tests were performed in R to identify the ontology groups enriched in the proteins differentially expressed under the two conditions tested.

At late stages of growth, a population of S. aureus exhausts glucose and expression of TCA cycle enzymes is upregulated (6). These conditions emulate the nutrient environment present during S. aureus infection (6). Proteome analysis confirms that this is the case in a stationary-phase culture, where the level of TCA cycle enzymes increases whereas the abundance of glycolytic enzymes decreases. High levels of enzymes responsible for incorporating amino acids in TCA cycle metabolism are also observed in stationary-phase cells (Fig. 1; see Table S1 in the supplemental material). The stationary phase is characterized by nutrient depletion, which accounts for previously observed low levels of ATP and high tolerance of antibiotics (5). In a late exponentially growing population where oxygen is available but glucose has been largely exhausted, fluctuation in the levels of TCA cycle enzymes could then lead to a drug-tolerant state, characterized by a characteristic increase in persisters. We first sought to examine this in a model experiment by testing antibiotic tolerance of mutants with knockouts in TCA cycle enzymes.

**FIG 2:**
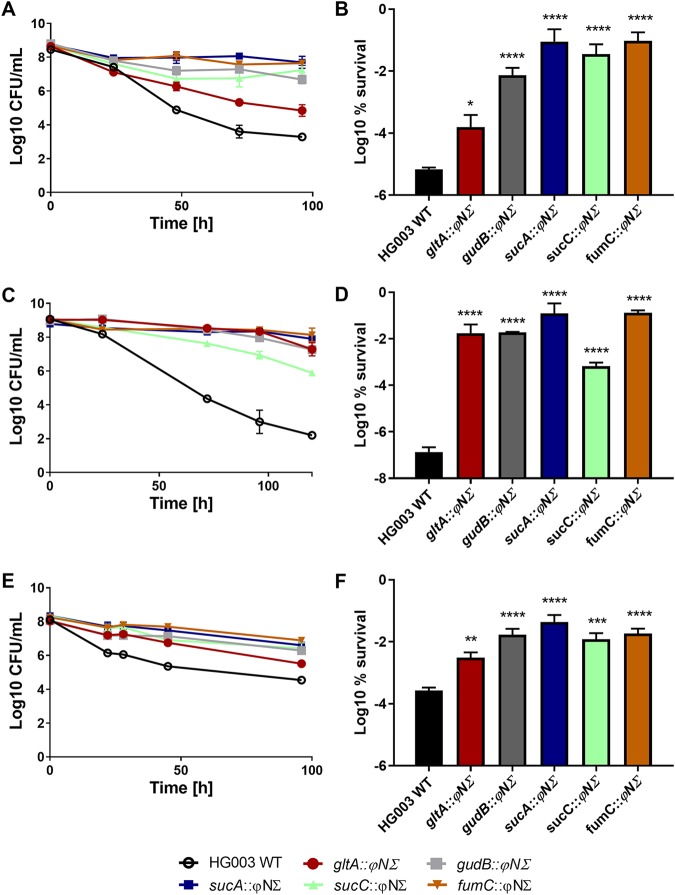
S. aureus lacking functional late TCA cycle genes exhibits increased antibiotic tolerance. (A, C, and E) Data represent levels of antibiotic killing of mutants with mutations in TCA cycle genes *gltA*, *sucA*, *sucC*, *fumC*, and *gudB* (encoding citrate synthase, 2-oxogluterate dehydrogenase, succinyl coenzyme A synthetase, fumarase, and glutamate dehydrogenase, respectively) over time compared to the wild type (open symbols) after 5 h of growth and 10× MIC antibiotic challenge in TSB medium. (B, D, and F) Bar graph data represent percent survival of each strain at the end of each treatment. (A and B) Ciprofloxacin treatment. (C and D) Gentamicin treatment. (E and F) Oxacillin treatment. Error bars indicate standard errors of the means (SEM). Asterisks indicate the statistical significance of results of comparisons between a mutant and the wild type as determined by Sidak’s multiple-comparison test (*, *P* < 0.05; **, *P* < 0.01; ***, *P* < 0.001; ****, *P* < 0.0001). Experiments were performed in biological triplicate.

We constructed mutants lacking representative functional enzymes in early, *gltA* (citrate synthase) TCA cycle enzymes and in late, *gudB* (glutamate dehydrogenase), *sucA* (2-oxoglutarate dehydrogenase), *sucC* (succinyl-coenzyme A [CoA] synthetase), *fumC* (fumarate hydratase) TCA cycle enzymes by transducing insertions from the Nebraska Transposon Mutant Library into methicillin-susceptible S. aureus (MSSA) strain HG003, which is susceptible to antibiotics (7). The mutants did not exhibit a growth defect. After 5 h of growth, all strains reached the same CFU level as the wild type (WT) (Fig. 2). Strains grown to the late exponential phase were challenged with 10× MIC of ciprofloxacin, gentamicin, or oxacillin, representing the main classes of bactericidal antibiotics—fluoroquinolones, aminoglycosides, and β-lactams (see Table S2 for the MICs for each strain). The TCA cycle and amino acid catabolism are important for S. aureus growth *in vivo*, so we also investigated glutamate dehydrogenase. Glutamate dehydrogenase fuels the TCA cycle under glucose-depleted conditions by converting glutamate derived from the abundant proline present in collagen into 2-oxoglutarate (8). All TCA cycle mutants as well as the *gudB* strain had significantly higher survival than the wild-type strain upon treatment with antibiotics, showing the multidrug-tolerant phenotype characteristic of persisters (Fig. 2). The highest level of persisters is observed with *sucA* and *fumC* mutants, where nearly 10% of the population consists of persisters.

**FIG 3:**
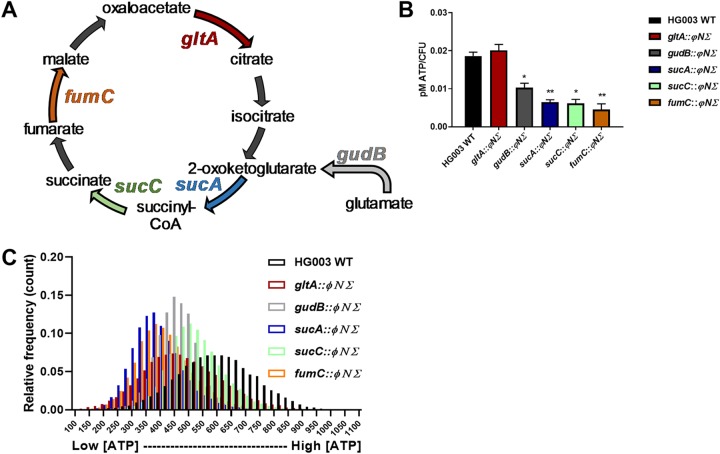
TCA cycle mutant strains have lower ATP levels than wild-type strains. (A) TCA cycle model. The mutants are highlighted in bold. (B) ATP concentration per CFU of all strains. Cultures were grown to the late exponential phase, and ATP levels were measured in the bulk population by luminescence assay. Mutants *gudB*, *sucA*, *sucC*, and *fumC* had significantly lower ATP levels than the wild-type strain. Error bars represent standard errors. Statistical significance was determined using analysis of variance (ANOVA) followed by Sidak’s multiple-comparison test (*, *P* < 0.05; **, *P* < 0.01). (C) Distribution of frequencies of QUEEN signal for each TCA mutant and wild-type strain. Wild-type S. aureus and TCA mutants were grown to the early stationary phase in TSB without glucose at 30°C. Strains were grown with chloramphenicol 10 μg/ml to maintain the plasmid, and QUEEN expression was induced with 0.03% xylose. Single cells were analyzed by flow cytometry. Postacquisition analysis was performed with FlowJo software. Ratio values were calculated by dividing the intensity values determined for excitations performed at 405 nm and 488 nm (405ex/488ex).

We reasoned that strains with high persister levels should have low ATP levels. We measured ATP levels with luciferase and observed that *gudB*, *sucA*, *sucC*, and *fumC* mutants indeed had significantly lower ATP levels than the wild-type strain in late exponential phase (Fig. 3B). Low membrane potential, rather than ATP, has been recently proposed as the cause of persister formation (9). We measured the membrane potential of the TCA cycle mutants and found that changes in ATP levels correlated better with cell survival upon antibiotic treatment than membrane potential did (see Fig. S1 in the supplemental material).

10.1128/mBio.01930-19.1 FIG S1 Membrane potential and ATP correlation. Data in correlation plots represent percent survival after antibiotic treatment with ATP (A, C, and E) and membrane potential (B, D, and F). (G) Quantification of membrane potential. A total of 4,721 cells were subjected to membrane potential analysis. Download FIG S1, DOCX file, 0.1 MB. Copyright © 2019 Zalis et al. 2019 Zalis et al. This content is distributed under the terms of the Creative Commons Attribution 4.0 International license.

In order to probe the heterogeneity of the population, we sought to examine ATP at the single-cell level. For this, we used the QUEEN ATP sensor (10). QUEEN contains green fluorescent protein (GFP) fused to an ATP-binding subunit of *Bacillus* PS3 F0F1 ATP synthase. The sensor absorbs at 405 nm and 488 nm and emits at 513 nm. At higher levels of ATP, there is increased fluorescence under conditions of 405-nm excitation and decreased fluorescence under conditions of 488-nm excitation. A ratio between the two emission signals reports ATP concentration. This ratio does not depend on the amount of the reporter, eliminating errors due to variations in QUEEN levels among cells. We first cloned QUEEN in plasmid pEPSA5 under the control of a xylose promoter, but the fluorescence signal was weak. We then performed codon optimization of a QUEEN construct for expression in S. aureus, which yielded an improved fluorescence signal (Fig. S2).

10.1128/mBio.01930-19.2 FIG S2 Optimization of *QUEEN2m* for expression in S. aureus. Images are representative of exponential-phase cultures of S. aureus HG003-pEPSA5-*QUEEN2m* and HG003-pEPSA5-*QUEEN2m*(opt) cultured at 30°C in TSB without glucose (complemented with chloramphenicol 10 μg/ml and xylose 0.03% for maintenance of pEPSA5 and induction of *QUEEN2m* expression, respectively). Samples were washed with PBS, placed on top of a PBS–1% agarose pad, and observed under the microscope. The two fluorescent signals corresponding to 405ex (false-colored here in magenta) and 488ex (false-colored here in green) were sequentially collected one after the other. Scale bar, 5 μm. Download FIG S2, DOCX file, 0.8 MB. Copyright © 2019 Zalis et al. 2019 Zalis et al. This content is distributed under the terms of the Creative Commons Attribution 4.0 International license.

Using fluorescence-activated cell sorter (FACS) analysis, we monitored ATP in single cells of wild-type S. aureus and the *gltA*, *gudB*, *sucA*, *sucC*, and *fumC* mutants. The frequency distribution of the ratios is shown in Fig. 3C. A wide range of distributions of ATP among cells is evident both in the wild-type population and in the TCA cycle mutants (Fig. 3C). As expected, the distribution of ATP in mutant populations was shifted to lower levels than were seen with the wild type, consistent with an increase in the levels of persisters. Notably, the overall level of ATP in a knockout in the early TCA cycle enzyme encoded by *gltA* was similar to that of the wild type (Fig. 3B), while survival in the presence of antibiotics was increased compared to the wild type (Fig. 2). However, the distribution of ATP in the population clearly shows a significant fraction of cells with lower ATP levels (Fig. 3C), explaining the higher level of tolerance of this mutant.

Persisters tend to wake up during sorting, which makes sorting prior to antibiotic treatment problematic (2, 5). Sorting based on the QUEEN signal after antibiotic treatment would not report the ATP status of cells immediately prior to treatment. Given that knockouts in TCA cycle had lower ATP levels, we used reporters of their expression to identify low-energy cells. Adding antibiotic prior to sorting provides a snapshot of a protein level at that point in time.

**FIG 4:**
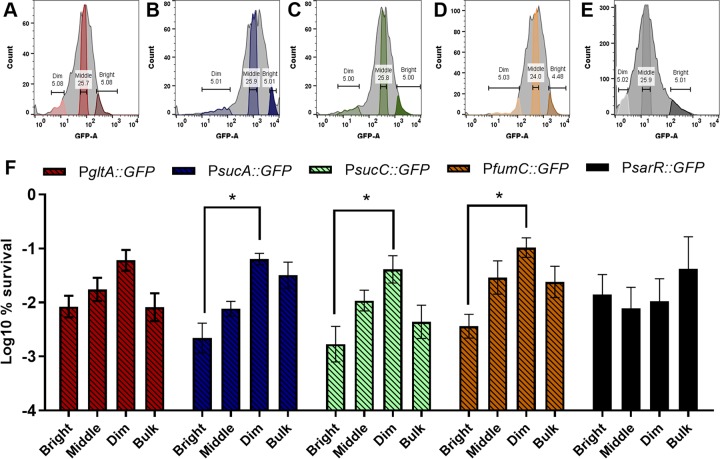
Sorting of cells with low levels of expression of TCA cycle enzymes enriches in drug-tolerant persisters. (A to E) GFP expression of P*gltA*::*gfp*, P*sucA*::*gfp*, P*sucC*::*gfp*, P*fumC*::*gfp*, and *PsarR*::*gfp*. (F) Percent survival of each gated fraction and the bulk of the unsorted population. Cells with low levels of expression (Dim) of *sucA*, *sucC*, and *fumC* exhibited significantly increased survival compared to cells with relatively high expression (Bright). Sorting on the basis of constitutively expressed P*sarR*::*gfp* yielded no significant enrichment in any fraction. Asterisks indicate statistical significance as determined by two-way ANOVA in multiple comparisons (*, *P* < 0.05). The graph presents means of results from five biological replicates. A representative plate is shown in Fig. S3.

We cloned the promoter regions of TCA cycle genes upstream of *gfp_uvr_* in plasmid pALC1434 (11), yielding P*gltA*::*gfp*, P*sucA*::*gfp*, P*sucC*::*gfp*, and P*fumC*::*gfp*. These strains were grown to the late exponential phase and challenged with ciprofloxacin at 10× MIC. After 24 h of antibiotic treatment, cells were subjected to FACS analysis (Fig. 4). Interestingly, there was a broad distribution of expression levels for each of these genes in the population observed before and after antibiotic treatment. Populations with low, intermediate, and high (“dim, middle, and bright”) TCA gene expression levels were gated (Fig. 4A to D) and sorted onto agar plates. The surviving cells formed colonies, and we quantified the level of survival of each gated fraction of cells compared to the bulk of the population. We observed a significant enrichment in persister cells in the dim fractions. In the cases of *sucA*, *sucC*, and *fumC*, there was a nearly 100-fold difference in the levels of persisters between the dim and bright fractions (Fig. 4). In order to test for a possible correlation between persister levels and a general decrease in transcription, we performed an identical experiment with a strain containing a reporter of the constitutively expressed *sarR* gene. There were no differences in persister levels among the dim, middle, and bright populations of the control. Taken together, these results suggest that random fluctuations in the levels of TCA cycle enzymes cause a decrease in the energy level, producing drug-tolerant persisters.

10.1128/mBio.01930-19.3 FIG S3 Representative photograph of colonies formed by surviving cells after antibiotic challenge and sorting. Data represent results of FACS analysis of P*sucA*::*gfp* gated into dim, middle, and bright fractions. Here, 1,600 cells were sorted per fraction. Download FIG S3, DOCX file, 0.1 MB. Copyright © 2019 Zalis et al. 2019 Zalis et al. This content is distributed under the terms of the Creative Commons Attribution 4.0 International license.

## DISCUSSION

Persisters were originally discovered in a study of S. aureus in 1944 (12, 13), but understanding the mechanism of their formation proved to be unusually challenging (12, 14). This no doubt is due to the small size of their population and a temporary phenotype. Given that bactericidal antibiotics act by corrupting their targets, we proposed that tolerance is caused by target inactivation (2, 5). This concept is broad enough to cover the two types of emerging persister formation mechanisms, i.e., the specific and the generalized. Specialized toxins such as HipA and TisB govern specific mechanisms of persister formation. Selection for high-persister-level mutants led to the identification of a gain-of-function allele in the *hipA* mutant in E. coli (15), and subsequent studies determined that it is a kinase (16) that inhibits translation by phosphorylating glu-tRNA synthase (17, 18). However, deletion of the *hipBA* locus has no phenotype, and it does not appear that HipA plays a role in persister formation of wild-type E. coli. At the same time, we found that *hipA7* high-persister-level cells were present in E. coli isolates from patients with urinary tract infection, a result of *in vivo* selection for drug tolerance (19). Another E. coli toxin, TisB, provides an example of a persister formation mechanism operating in wild-type cells. Induced by fluoroquinolones through the SOS DNA damage response, TisB is an endogenous antimicrobial peptide that causes tolerance by decreasing the levels of proton motive force (PMF) and ATP (20, 21). However, these specific mechanisms do not explain how persisters are formed under regular growth conditions. For a while, the idea that RNA endonuclease TAs such as RelBA or MazEF constitute the main mechanism of persister formation in bacteria represented the standard model (22), but several recent studies failed to find a connection between these interferase toxins and persisters in E. coli (2–5). In particular, a knockout of 10 interferase TAs had no effect on persister formation (3, 4).

In search of a general mechanism of persister formation, we identified a link between low energy, specifically, low ATP levels, and drug tolerance in both S. aureus and E. coli (2, 5). Selectively decreasing the level of ATP by arsenate is sufficient to produce persisters, and low-energy cells sorted from a population by monitoring transcriptional or translational ATP markers are tolerant of antibiotics (2, 5). If ATP levels are low, target activity is diminished, providing a simple mechanism for drug tolerance. In the current study, we sought to identify components that are responsible for producing persisters. Energy-generating components are a logical choice to consider, and, not surprisingly, we observed, in agreement with previous observations (9), that knocking out TCA cycle components increased drug tolerance and lowers ATP levels. The critical issue, however, is whether natural fluctuations in the levels of expression of TCA enzymes are sufficient to produce persisters. We found that sorting cells with low levels of expression of several TCA cycle enzymes encoded by *gltA*, *sucA*, *sucC*, and *fumC* resulted in enrichment of drug-tolerant persisters. Interestingly, FACS analysis showed that the level of noise in expression of these enzymes is considerable, ranging over 3 orders of magnitude, and largely follows a typical Gaussian distribution. This noise apparently leads to formation of rare cells with low levels of TCA cycle enzyme expression and decreased levels of ATP and of drug tolerance.

In retrospect, the low-energy hypothesis of persister formation is quite obvious—indeed, the simplest way to inactivate all antibiotic targets is by reducing ATP levels, and the specialized pore-forming TisB (20, 23) and HokB (24) toxins provide a precedent for this. However, the idea that the noise in an energy-generating component is sufficient to produce a drop in ATP levels is counterintuitive. It is commonly accepted that noise results from fluctuations in the levels of expression of sparsely expressed components. In a classical example, E. coli cells have only about 10 molecules of LacI on average, and noise in expression produces rare cells with no repressor, resulting in full expression of the *lac* operon in the absence of inducer (25). In contrast, TCA cycle enzymes are abundant, and the considerable level of noise in their expression that we observed was unexpected.

The current report represents the first step toward identifying components that can lead to a low energy state and drug tolerance. Future studies will show how widely spread among bacteria is this general mechanism of persister formation.

## MATERIALS AND METHODS

### Bacterial strains, culture conditions, and strain construction.

S. aureus strains were grown in tryptic soy broth (TSB) (MP Biomedicals, USA) or in TSB without glucose (Becton, Dickinson, and Company, USA) or in Mueller-Hinton broth (MHB) as indicated. Cultures were grown at 37°C with shaking at 225 rpm. Strains encoding the QUEEN construct were grown on tryptic soy agar (TSA) plates with chloramphenicol 10 μg/ml at 30°C or in TSB without glucose with shaking at 225 rpm at 30°C with chloramphenicol 10 μg/ml to maintain the pEPSA5 plasmid. QUEEN expression was induced with 0.03% xylose. High concentrations of xylose led to a growth defect. MSSA strain HG003 was used for these studies, and the mutations for all TCA cycle genes in this background were transduced from the Nebraska Transposon Mutant Library (NTML) using bacteriophage 80α or φ11. Mutations were subsequently confirmed by amplification of the sequence from the beginning or end of the gene of interest to the transposon insertion as previously described (7). For construction of *gfp* reporters, promoter regions of *gltA*, *sucAB*, *sucC*, or *fumC* were cloned upstream of *gfp* into the EcoRI and XbaI sites of pALC1434 (11). P*sucAB* was amplified with 5′-GGGCCCGAATTCGAAACCTCATCAATTCGAACAA-3′ and 5′-GGGCCCTCTAGATTTACACCCTCCACAAAAATGTTGAAA-3′. Additional data are available in Table S2 in the supplemental material.

10.1128/mBio.01930-19.4 TABLE S1 Details of proteins and enrichment represented in the map in Fig. 1. *P* value was determined by ANOVA. Download Table S1, DOCX file, 0.03 MB. Copyright © 2019 Zalis et al. 2019 Zalis et al. This content is distributed under the terms of the Creative Commons Attribution 4.0 International license.

10.1128/mBio.01930-19.5 TABLE S2 MICs for all TCA cycle mutants and wild-type strains. MICs were determined using the broth microdilution method. All values are in μg/ml. Download Table S2, DOCX file, 0.02 MB. Copyright © 2019 Zalis et al. 2019 Zalis et al. This content is distributed under the terms of the Creative Commons Attribution 4.0 International license.

Escherichia coli DH5α was used to propagate plasmids. DH5α strains were grown in LB broth, Miller (Fisher BioReagents, USA), and ampicillin 100 μg/ml was used to maintain plasmids where necessary.

### Proteomic sample preparation.

Four replicates were prepared from either exponential-phase or stationary-phase cells, and cells were grown for 4 or 24 h, respectively, after overnight cultures were diluted 1:1,000 in 100 ml MHB. Cells were grown in 500-ml baffled flasks at 37°C with shaking at 225 rpm. Cells were pelleted at 5,000 × *g* for 7 min at 4°C, washed twice in phosphate-buffered saline (PBS), and pelleted again. Pellets were resuspended in 500 μl PBS, transferred to a 2-ml tube, washed a final time, and then flash frozen with liquid nitrogen and stored at –80°C. Bacterial pellets were resuspended in 100 mM ammonium bicarbonate and lysed by vortex mixing 5 times for 1 min and then with 0.1-mm-diameter zirconia/silica beads with resting periods of 30 s on ice. Samples were then digested with trypsin as previously described (26) and desalted using C_18_ solid-phase-extraction (SPE) cartridges (Discovery C_18_; Supelco) (1 ml, 50 mg). Peptide concentrations were measured by bicinchoninic acid (BCA) assay (Thermo Scientific).

### Proteomics and data analysis.

For each sample, a 0.5-μg volume of peptides was separated using a 200-min gradient and a Waters nanoACQUITY ultraperformance liquid chromatography (UPLC) system (Millford, MA) coupled with a Q Exactive HF mass spectrometer (MS; Thermo Fisher Scientific). MS scans were recorded at a resolution of 35,000. The top 12 ions from the survey scan were selected by the use of a quadrupole mass filter for high-energy collision dissociation, and mass data were analyzed by the use of an Orbitrap analyzer. A window of 2 *m*/*z* was used for the isolation of ions with a collision energy level of 28%. Tandem MS (MS/MS) spectra were recorded at a resolution of 17,500. The resulting data were processed using MaxQuant V1.5.2.8. (27). Proteins were identified with at least 2 peptides of length greater than 6 residues. The RefSeq Staphylococcus aureus *NCTC8325* database was used for the search (July 2017; 2,768 sequences). Matches between runs and MaxLFQ data were used for quantification; other parameters were conserved by default. Proteins identified only by site, reverse hits, and contaminants were removed. Only protein groups with a measured label-free-quantitation (LFQ) intensity level in at least 60% of the examples of one sample type were conserved for further quantification. LFQ intensities were log2 transformed and median normalized. Analyses of statistics (e.g., Student’s *t* test, scaling, etc.) and normalization steps were performed in R using the Stat packages.

### Persister assays.

Overnight cultures were diluted 1:1,000 in 2 ml TSB (Fisher, MP Biomedicals) in a 14-ml capped culture tube (VWR International) and grown 5 h to the late exponential phase, and starting CFU were plated. Details regarding the proteins identified and the enrichment can be found in Table S1 in the supplemental material. Cultures were challenged with ciprofloxacin, oxacillin, or gentamicin (4, 1.25, and 100 μg ml^−1^, respectively). To enumerate survivors over time, 100 μl of culture was removed, pelleted by centrifugation, washed with 1% NaCl, serially diluted, and plated on TSA, and CFU counting was performed after 24 h of regrowth on TSA to enumerate survivors. Experiments were performed in biological triplicate.

### ATP quantification of bulk culture.

ATP levels were measured using the Promega BacTiter-Glo microbial cell viability assay according to the manufacturer’s instructions. A working volume of 100 μl was used. Aliquots were removed from tubes, pelleted, and resuspended in 1% NaCl before reading luminescence. Experiments were performed in biological triplicate.

### Construction of S. aureus HG003 expressing *QUEEN2m*.

**(i) pEPSA5-*QUEEN2m*.** pEPSA5-*QUEEN2m* was created by amplifying *QUEEN2m* from pRsetB-his7-*QUEEN2m* (10) with primers Q2m-F (5′-CGAGCTGAATTC**TAGGGAGAGGTTTTAAAC**ATGAAAACTGTGAAAGTGAATATAAC-3′) and Q2m-R (5′-CGAGCTGGTACCTCACTTCATTTCCGCAACGCTC-3′) and cloning it into the EcoRI/KpnI sites of pEPSA5 downstream of a xylose promoter (28). Restrictions sites are underlined, and a ribosome binding site (RBS) from *sarA* gene has been added in Q2m-F primer (indicated here in bold).

**(ii) pEPSA5-*QUEEN2m*(opt).** *QUEEN2m*(opt) was codon optimized from the original sequence by using the JCat tool (29), and DNA was synthesized (Genewiz) with the same RBS as was used with pEPSA5-*QUEEN2m* in a custom plasmid. A fragment containing *QUEEN2m*(opt) and its RBS was excised from this plasmid with EcoRI/KpnI and cloned into the EcoRI/KpnI sites of pEPSA5 (28).

Those two plasmids were transformed into S. aureus RN4220 and amplified from this background before being transformed in the S. aureus HG003 background. Only the optimized version was transformed into mutant strains.

### Microscopy.

S. aureus HG003-pEPSA5-*QUEEN2m* and HG003-pEPSA5-*QUEEN2m*(opt) were cultured at 30°C in TSB without glucose (complemented with chloramphenicol 10 μg/ml and xylose 0.03% for maintenance of pEPSA5 and induction of *QUEEN2m* expression, respectively) to the stationary phase, inoculated into fresh medium of the same composition at 1:100, and cultured for 4.5 h at 30°C. Samples were washed with PBS, placed on top of a PBS–1% agarose pad, and observed under a Zeiss LSM 710 confocal microscope using a 63× oil immersion lens objective. The two fluorescent signals, 405ex and 488ex, were sequentially collected. Differential interference contrast (DIC) images were recorded alongside the 405ex acquisition. Images were acquired by the use of Zen software at a resolution of 1,024 by 1,024 pixels, with a lane average of 8, and were processed with Fiji software (30).

### Single-cell ATP quantification using QUEEN.

Flow cytometry was carried out using an Attune flow cytometer. Spatially separated violet and blue lasers were used for excitation at 405 nm and 488 nm, respectively, to calculate the ratio of the level produced at each of these excitation wavelengths to that seen with emission at 513 nm. To prepare samples, –80°C stocks were grown on TSA plates with chloramphenicol 10 μg/ml at 30°C. Single colonies were selected, and overnight cultures were inoculated into TSB without glucose plus chloramphenicol 10 μg/ml with shaking at 225 rpm at 30°C. Tubes were inoculated 24 h later from overnight cultures and grown for 25 h (HG003 empty vector; HG003 WT; *gltA*, *sucA*, and *fumC* mutants) or 26 h (*gudB* and *sucC* mutants) to reach comparable levels of CFU per milliliter. Xylose 0.03% was used to induce expression of QUEEN. The strains used was as follows: HG003 pEPSA5 (empty vector), HG003 pEPSA5-*QUEEN2m*, *gltA*::*ϕΝΣ* pEPSA5-*QUEEN2m*, *gudB*::*ϕΝΣ* pEPSA5-*QUEEN2m*, *sucA*::*ϕΝΣ* pEPSA5-*QUEEN2m*, *sucC*::*ϕΝΣ* pEPSA5-*QUEEN2m*, and *fumC*:: *ϕΝΣ* pEPSA5-*QUEEN2m*. Three biological replicates were analyzed for each strain.

### FACS analysis using GFP reporters.

Cell sorting was carried out using a BD FACSAria II flow cytometer with a 70-μm-inner-diameter nozzle. Briefly, 1:1,000 dilutions of overnight cultures were grown 5 h to late exponential phase and challenged with ciprofloxacin (10× MIC) for 24 h. After 24 h, cells were diluted 1:100 and sonicated as previously described (31) to disperse aggregates of S. aureus cells. FACS DIVA software was used in sorting setup; the initial population of cells was gated by size using forward scatter (FSC) and side scatter (SSC) and then on the basis of GFP fluorescence (GFP-A). Gates were set to include the brightest 5% and dimmest 5%, in addition to a middle level (∼30% of the population). Cells were sorted from each population onto TSA plates. Plates were incubated at 37°C for 24 h, and colonies were counted. Recorded colonies were normalized for expected colony counts by the use of an untreated control sort plate. Percent survival was calculated from the dim, middle, and bright GFP fractions.

### Membrane potential.

Membrane potential was measured using a *Bac*Light bacterial membrane potential kit according to the manufacturer’s instructions. Briefly, cultures were grown 5 h in TSB medium and diluted to 10^6^ CFU/ml in 1 ml filtered PBS. DiOC_2_(3) (3,3′-dihexyloxacarbocyanine iodide) was used to stain cells for 30 min before samples were analyzed by flow cytometry. The ratio of mCherry fluorescence to GFP fluorescence was calculated for each cell. The negative-control sample was pretreated with carbonyl cyanide *m*-chlorophenylhydrazone (CCCP) for 5 min before staining was performed. For flow cytometry analysis, cells were gated by size, and then ratios were calculated.

### Data availability.

The mass spectrometry proteomics data have been deposited in the ProteomeXchange Consortium with the data set identifier PXD013151 and on MassIVE under the identifier MSV000083600 (ftp://massive.ucsd.edu/MSV000083600/).
