# Supplementary figures and images for: Stochastic Variation in Expression of the Tricarboxylic Acid Cycle Produces Persister Cells
Source: mBio. 2019 Sep 17;10(5):e01930-19. doi: 10.1128/mBio.01930-19 (PMC6751062; doi:10.1128/mBio.01930-19)

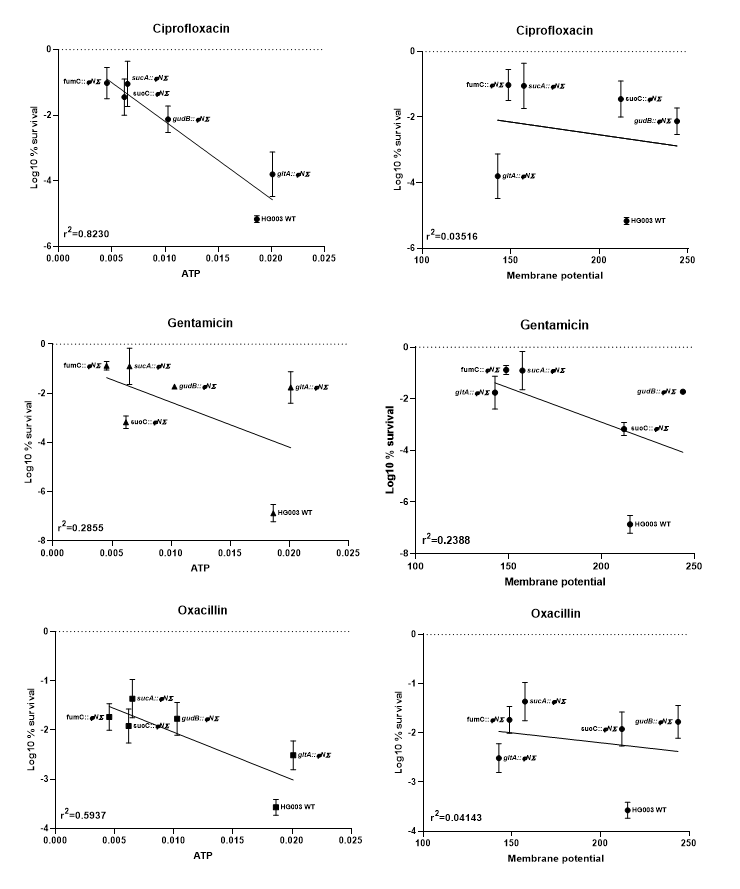


**B**

**A**

**D**

**C**

**E**

**F**


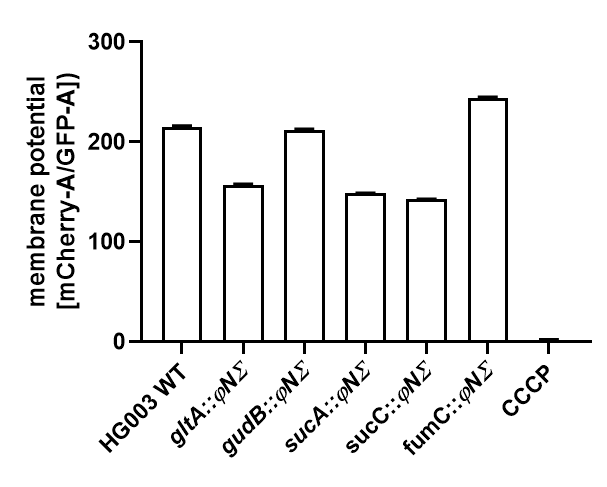


**G**

Supplement: FIG S1 [file mBio.01930-19-sf001.docx]

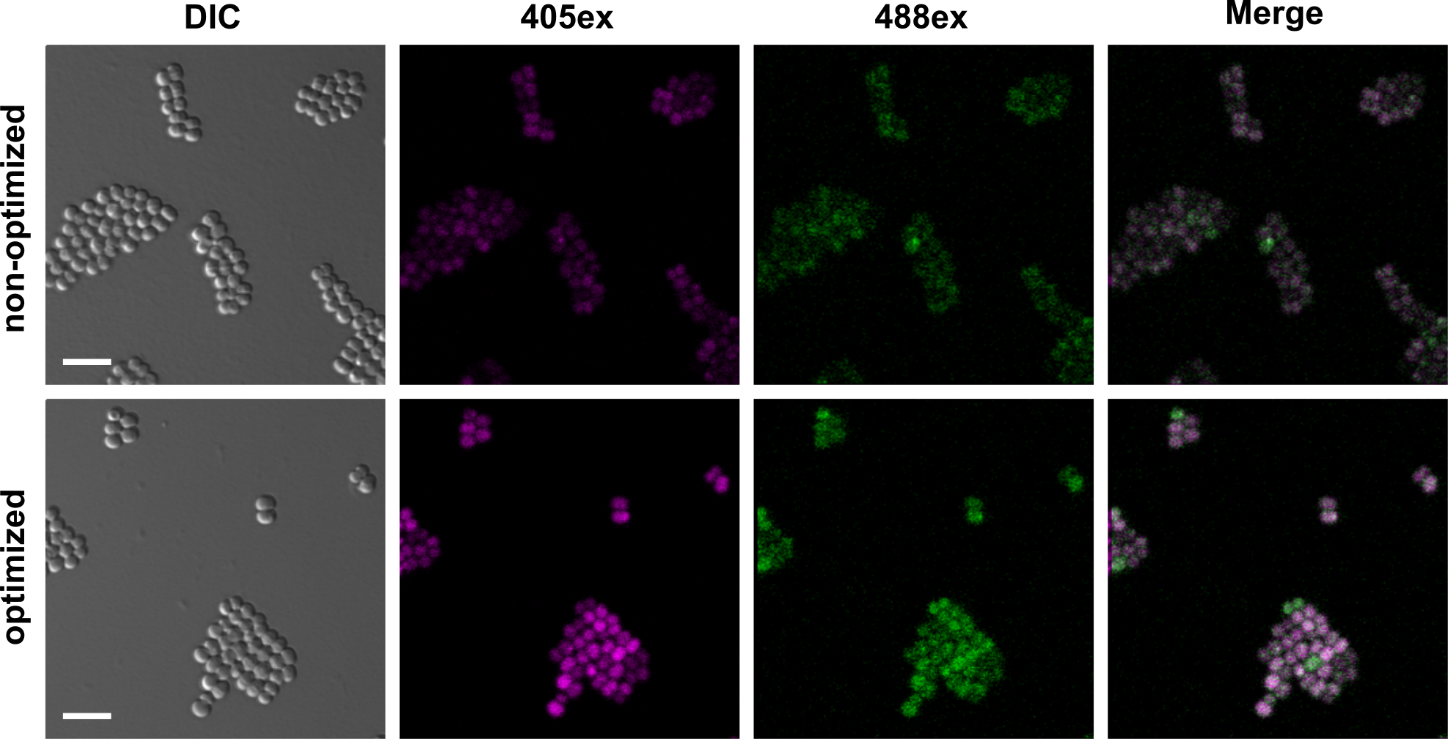

Supplement: FIG S2 [file mBio.01930-19-sf002.docx]
