# Supplementary material for: Stochastic Variation in Expression of the Tricarboxylic Acid Cycle Produces Persister Cells
Source: mBio. 2019 Sep 17;10(5):e01930-19. doi: 10.1128/mBio.01930-19 (PMC6751062; doi:10.1128/mBio.01930-19)
Supplement: FIG S3 [file mBio.01930-19-sf003.docx]

Dim

Middle

Bright
