# Supplementary material for: Stochastic Variation in Expression of the Tricarboxylic Acid Cycle Produces Persister Cells
Source: mBio. 2019 Sep 17;10(5):e01930-19. doi: 10.1128/mBio.01930-19 (PMC6751062; doi:10.1128/mBio.01930-19)
Supplement: TABLE S1 [file mBio.01930-19-st001.docx]

| **gene symbol** | **refSeq ID** | **Description** | **UniprotID** | | **Locus** | **pvalue** | **log2 (stat/exp)** | **Pathway** | **E.C. number** |
| --- | --- | --- | --- | --- | --- | --- | --- | --- | --- |
| ptsG | YP_501305.1 | PTS system glucose-specific transporter subunit IIABC | | PTU3C_STAA8 | SAOUHSC_02848 | **0.000518** | -0.7 | Glycolysis | 2.7.1.199 |
| glcA | YP_498754.1 | PTS system glucose-specific protein | | PTG3C_STAA8 | SAOUHSC_00155 | **0.000218** | -0.7 | Glycolysis | 2.7.1.199 |
| ptbA | YP_499955.1 | PTS system transporter subunit IIA | | Q2FYL0_STAA8 | SAOUHSC_01430 | **0.004213** | 0.5 | Glycolysis | 2.7.1.199 |
| pgi | YP_499453.1 | Glucose-6-phosphate isomerase | | G6PI_STAA8 | SAOUHSC_00900 | **0.000144** | -1.0 | Glycolysis | 5.3.1.9 |
| pfkA | YP_500312.1 | 6-phosphofructokinase | | PFKA_STAA8 | SAOUHSC_01807 | **0.000041** | -1.5 | Glycolysis | 2.7.1.11 |
| FBPase | YP_501280.1 | hypothetical protein SAOUHSC_02822 | | F16PC_STAA8 | SAOUHSC_02822 | 0.928360 | 0.0 | Glycolysis | 3.1.3.11 |
| fdaB | YP_501379.1 | fructose-1,6-bisphosphate aldolase | | ALF1_STAA8 | SAOUHSC_02926 | **0.006561** | -0.4 | Glycolysis | 4.1.2.13 |
| fbaA | YP_500842.1 | fructose-bisphosphate aldolase | | Q2FWD3_STAA8 | SAOUHSC_02366 | **0.006795** | -0.4 | Glycolysis | 4.1.2.13 |
| tpiA | YP_499353.1 | triosephosphate isomerase | | TPIS_STAA8 | SAOUHSC_00797 | **0.000336** | -1.0 | Glycolysis | 5.3.1.1 |
| gap | YP_499351.1 | glyceraldehyde-3-phosphate dehydrogenase | | Q2G032_STAA8 | SAOUHSC_00795 | **0.000001** | -1.3 | Glycolysis | 1.2.1.12 |
| gapB | YP_500298.1 | glyceraldehyde 3-phosphate dehydrogenase 2 | | Q2FXP2_STAA8 | SAOUHSC_01794 | 0.050871 | -0.7 | Glycolysis | 1.2.1.12 |
| pgk | YP_499352.1 | phosphoglycerate kinase | | PGK_STAA8 | SAOUHSC_00796 | **0.000196** | -1.1 | Glycolysis | 2.7.2.3 |
| gpmA | YP_501165.1 | phosphoglyceromutase | | GPMA_STAA8 | SAOUHSC_02703 | **0.000628** | -0.9 | Glycolysis | 5.4.2.11 |
| pgmB | YP_498949.1 | phosphoglycerate mutase family protein | | Q2G101_STAA8 | SAOUHSC_00359 | 0.094929 | -9.8 | Glycolysis | 5.4.2.11 |
| pgm | YP_499352.1 | phosphoglycerate kinase | | PGK_STAA8 | SAOUHSC_00796 | **0.000196** | -1.1 | Glycolysis | 5.4.2.11 |
| eno | YP_499355.1 | phosphopyruvate hydratase | | ENO_STAA8 | SAOUHSC_00799 | **0.000014** | -0.7 | Glycolysis | 4.2.1.11 |
| pykA | YP_500311.1 | pyruvate kinase | | KPYK_STAA8 | SAOUHSC_01806 | **0.000170** | -1.2 | Glycolysis | 2.7.1.40 |
| lctE | YP_498803.1 | L-lactate dehydrogenase | | LDH1_STAA8 | SAOUHSC_00206 | 0.048359 | -11.5 | Pyruvate/acetate metabolism | 1.1.1.27 |
| ldh2 | YP_501374.1 | L-lactate dehydrogenase | | LDH2_STAA8 | SAOUHSC_02922 | **0.000452** | -0.6 | Pyruvate/acetate metabolism | 1.1.1.27 |
| ddh | YP_501289.1 | D-lactate dehydrogenase | | Q2FVA3_STAA8 | SAOUHSC_02830 | **0.000300** | -1.4 | Pyruvate/acetate metabolism | 1.1.1.28 |
| pycA | YP_499610.1 | pyruvate carboxylase | | Q2G2C1_STAA8 | SAOUHSC_01064 | 0.390606 | -0.2 | Pyruvate/acetate metabolism |  |
| pdhA | YP_499589.1 | pyruvate dehydrogenase complex, E1 component subunit alpha | | Q2FZG4_STAA8 | SAOUHSC_01040 | **0.001604** | -1.2 | Pyruvate/acetate metabolism | 1.2.4.1 |
| pdhB | YP_499590.1 | pyruvate dehydrogenase complex, E1 component subunit beta | | Q2G2A5_STAA8 | SAOUHSC_01041 | **0.000066** | -0.8 | Pyruvate/acetate metabolism | 1.2.4.1 |
| pdhC | YP_499591.1 | branched-chain alpha-keto acid dehydrogenase subunit E2 | | Q2G2A4_STAA8 | SAOUHSC_01042 | **0.000075** | -1.1 | Pyruvate/acetate metabolism | 2.3.1.12 |
| pdhD | YP_499592.1 | dihydrolipoamide dehydrogenase | | Q2G2A3_STAA8 | SAOUHSC_01043 | **0.000517** | -0.7 | Pyruvate/acetate metabolism | 1.8.1.4 |
| lpdA | YP_500129.1 | dihydrolipoamide dehydrogenase | | Q2FY51_STAA8 | SAOUHSC_01614 | 0.016388 | -0.8 | Pyruvate/acetate metabolism | 1.8.1.4 |
| pflB | YP_498784.1 | formate acetyltransferase | | PFLB_STAA8 | SAOUHSC_00187 | **0.008447** | -1.8 | Pyruvate/acetate metabolism | 2.3.1.54 |
| porA | YP_499799.1 | hypothetical protein SAOUHSC_01266 | | Q2FZ05_STAA8 | SAOUHSC_01266 | 0.015238 | -0.5 | Pyruvate/acetate metabolism | 1.2.7.11 |
| porB | YP_499800.1 | 2-oxoglutarate ferredoxin oxidoreductase subunit beta | | Q2FZ04_STAA8 | SAOUHSC_01267 | 0.050590 | -0.3 | Pyruvate/acetate metabolism | 1.2.7.11 |
| eutD | YP_499142.1 | phosphotransacetylase | | Q2G0J0_STAA8 | SAOUHSC_00574 | **0.000371** | -1.2 | Pyruvate/acetate metabolism | 2.3.1.8 |
| acsA | YP_500351.1 | acetyl-CoA synthetase | | Q2G294_STAA8 | SAOUHSC_01846 | **0.002462** | 1.5 | Pyruvate/acetate metabolism | 6.2.1.1 |
| AcsA2 | YP_501382.1 | acetyl-CoA synthetase | | Q2FV14_STAA8 | SAOUHSC_02929 | **0.004396** | -0.4 | Pyruvate/acetate metabolism | 6.2.1.1 |
| ackA | YP_500325.1 | acetate kinase | | ACKA_STAA8 | SAOUHSC_01820 | **0.000091** | -1.2 | Pyruvate/acetate metabolism | 2.7.2.1 |
| acyP | YP_499933.1 | acylphosphatase | | ACYP_STAA8 | SAOUHSC_01406 | **0.000000** | +inf | Pyruvate/acetate metabolism | 3.6.1.7 |
| CidC | YP_501306.1 | pyruvate oxidase | | Q2FV86_STAA8 | SAOUHSC_02849 | **0.000004** | -1.4 | Pyruvate/acetate metabolism |  |
| aldH | YP_500632.1 | aldehyde dehydrogenase | | Q2FWX9_STAA8 | SAOUHSC_02142 | 0.013902 | 0.9 | Pyruvate/acetate metabolism | 1.2.1.3 |
| aldA | YP_500839.1 | aldehyde dehydrogenase | | ALD1_STAA8 | SAOUHSC_02363 | 0.950916 | 0.0 | Pyruvate/acetate metabolism | 1.2.1.3 |
| aldA2 | YP_498732.1 | aldehyde dehydrogenase | | ALDA_STAA8 | SAOUHSC_00132 | 0.028068 | 0.4 | Pyruvate/acetate metabolism | 1.2.1.3 |
| adh1 | YP_499171.1 | alcohol dehydrogenase | | ADH_STAA8 | SAOUHSC_00608 | **0.001173** | -1.4 | Pyruvate/acetate metabolism | 1.1.1.1 |
| pckA | YP_500411.1 | phosphoenolpyruvate carboxykinase | | PCKA_STAA8 | SAOUHSC_01910 | **0.006573** | 0.9 | TCA | 4.1.1.49 |
| pycA | YP_499610.1 | pyruvate carboxylase | | Q2G2C1_STAA8 | SAOUHSC_01064 | 0.390606 | -0.2 | TCA | 6.4.1.1 |
| citZ | YP_500307.1 | hypothetical protein SAOUHSC_01802 | | Q2FXN3_STAA8 | SAOUHSC_01802 | **0.005531** | 0.8 | TCA | 2.3.3.1 |
| citB | YP_499875.1 | aconitate hydratase | | Q2FYS9_STAA8 | SAOUHSC_01347 | **0.000406** | 0.7 | TCA | 4.2.1.3 |
| citC | YP_500306.1 | isocitrate dehydrogenase | | Q2FXN4_STAA8 | SAOUHSC_01801 | 0.022720 | 0.4 | TCA | 1.1.1.42 |
| sucA | YP_499944.1 | 2-oxoglutarate dehydrogenase E1 component | | ODO1_STAA8 | SAOUHSC_01418 | 0.017669 | 0.4 | TCA | 1.2.4.2 |
| pdhD | YP_499592.1 | dihydrolipoamide dehydrogenase | | Q2G2A3_STAA8 | SAOUHSC_01043 | **0.000517** | -0.7 | TCA | 1.8.1.4 |
| lpdA | YP_500129.1 | dihydrolipoamide dehydrogenase | | Q2FY51_STAA8 | SAOUHSC_01614 | 0.016388 | -0.8 | TCA | 1.8.1.4 |
| odhB | YP_499943.1 | dihydrolipoamide succinyltransferase | | ODO2_STAA8 | SAOUHSC_01416 | **0.007355** | 0.6 | TCA | 2.3.1.61 |
| porA | YP_499799.1 | hypothetical protein SAOUHSC_01266 | | Q2FZ05_STAA8 | SAOUHSC_01266 | 0.015238 | -0.5 | TCA | 1.2.7.3 |
| porB | YP_499800.1 | 2-oxoglutarate ferredoxin oxidoreductase subunit beta | | Q2FZ04_STAA8 | SAOUHSC_01267 | 0.050590 | -0.3 | TCA | 1.2.7.3 |
| sucD | YP_499754.1 | succinyl-CoA synthetase subunit alpha | | Q2FZ36_STAA8 | SAOUHSC_01218 | 0.066954 | 0.3 | TCA | 6.2.1.5 |
| sucC | YP_499753.1 | succinyl-CoA synthetase subunit beta | | SUCC_STAA8 | SAOUHSC_01216 | 0.268254 | 0.2 | TCA | 6.2.1.5 |
| sdhC | YP_499647.1 | succinate dehydrogenase cytochrome b-558 subunit | | Q2FZC9_STAA8 | SAOUHSC_01103 | **0.009117** | 1.1 | TCA | 1.3.5.4 |
| sdhA | YP_499648.1 | succinate dehydrogenase flavoprotein subunit | | Q2FZC8_STAA8 | SAOUHSC_01104 | **0.000264** | 0.8 | TCA | 1.3.5.4 |
| sdhB | YP_499649.1 | succinate dehydrogenase iron-sulfur subunit | | Q2FZC7_STAA8 | SAOUHSC_01105 | **0.001424** | 0.5 | TCA | 1.3.5.4 |
| fumC | YP_500480.1 | fumarate hydratase | | Q2FX94_STAA8 | SAOUHSC_01983 | **0.000045** | 0.7 | TCA | 4.2.1.2 |
| mqo1 | YP_501109.1 | malate:quinone oxidoreductase | | Q2FVQ5_STAA8 | SAOUHSC_02647 | **0.002638** | 0.6 | TCA | 1.1.5.4 |
| mqo2 | YP_501380.1 | malate:quinone oxidoreductase | | Q2FV16_STAA8 | SAOUHSC_27 | 0.019618 | -0.9 | TCA | 1.1.5.4 |
