# Supplementary material for: Stochastic Variation in Expression of the Tricarboxylic Acid Cycle Produces Persister Cells
Source: mBio. 2019 Sep 17;10(5):e01930-19. doi: 10.1128/mBio.01930-19 (PMC6751062; doi:10.1128/mBio.01930-19)
Supplement: TABLE S2 [file mBio.01930-19-st002.docx]

| MIC (µg/mL) | HG003 WT | gltA | gudB | sucA | sucC | fumC |
| --- | --- | --- | --- | --- | --- | --- |
| Ciprofloxacin | 0.5 | 0.5 | 0.5 | 0.5 | 0.5 | 0.5 |
| Gentamicin | 0.5 | 0.5 | 0.5 | 0.5 | 0.5 | 0.5 |
| Oxacillin | 0.156 | 0.156 | 0.156 | 0.156 | 0.156 | 0.156 |
